# Underreporting COVID-19: the curious case of the Indian subcontinent

**DOI:** 10.1017/S0950268820002095

**Published:** 2020-09-11

**Authors:** Raaj Kishore Biswas, Awan Afiaz, Samin Huq

**Affiliations:** 1 Transport and Road Safety (TARS) Research Centre, School of Aviation, University of New South Wales, Sydney, New South Wales, Australia; 2 Institute of Statistical Research and Training, University of Dhaka, Dhaka, Bangladesh; 3 Child Health Research Foundation, Dhaka, Bangladesh

**Keywords:** Data validity, health system, pandemic, SARS-CoV-2, underreporting

## Abstract

COVID-19 has spread across the globe with higher burden placed in Europe and North America. However, the rate of transmission has recently picked up in low- and middle-income countries, particularly in the Indian subcontinent. There is a severe underreporting bias in the existing data available from these countries mostly due to the limitation of resources and accessibility. Most studies comparing cross-country cases or fatalities could fail to account for this systematic bias and reach erroneous conclusions. This paper provides several recommendations on how to effectively tackle these issues regarding data quality, test coverage and case counts.

Since the inception of the COVID-19 pandemic, both the media and research focus were on China, Europe and the USA primarily due to the large cluster of cases in these regions during the early days. However, despite low fatality rates and total cases, the focus then shifted to the low- and middle-income countries (LMICs) soon after the outbreak had hit the Indian subcontinent (ISC) and their COVID-19 response dynamics. In the meantime, academic studies started making inferences on the COVID-19 response effectiveness through comparing the disease prevalence and fatality rates between higher and lower income nations in order to investigate the curious case of low COVID-19 infection rates among the LMICs.

Conducting research on LMICs with limited data could often lead to erroneous findings and biased interpretations, which is becoming a concern with the avalanche of studies published daily. The reasons behind inadequate data in LMICs or even low fatality/detection rates could be qualitatively discussed. While these would not impede academicians in conducting research, caution should be exercised during interpretation and in proposals of ‘evidence-based’ policies and the development of operational plans focusing on mitigation and response in respective contexts. For a greater focus and authors' area of expertise, this paper is limited to the countries in the ISC, which is a sample representation of LMICs; however, country-wise discussion of LMICs based on the respective socioeconomic context is required for a reasonable generalisation.

## Rate and quality of testing

India, Pakistan and Bangladesh are among the worst 20 countries affected by the COVID-19 pandemic in terms of total number of cases; however, they are ranked 138, 139 and 147, respectively, in tests per million population, as of 18 June 2020 [1]. It is worth noting that countries such as Bangladesh reached the threshold of 10 000 tests per day on 20 May, an astonishing 74 days after the detection of the first confirmed case which was still a mere fraction of the number of tests conducted by countries such as the USA, UK and Italy. This lack of testing capabilities during the early days accompanied by the limited protective gears for health personnel and low implementation capacity related to the response of such pandemics could have concealed the true rate of infection and disease spread in the LMICs of the ISC.

Due to limited testing facilities and availability of trained personnel, long delays in both sample collection and dissemination of test results are observed in these countries [2, 3]. Furthermore, these inadequate facilities could compromise sample handling and storage as they require strict low temperature preservation for an optimum result [3], which is a challenge for LMICs including the ISC. Evidently, the official press releases in Bangladesh reflected that all collected samples were not tested daily with long backlogs leading to curbing sample collection [4] and resulting in public distress as many non-COVID medical facilities require certification of a negative test result before admitting new patients.

Another issue for maintaining a rigorous score of transmission rates is to adequately define both cases and deaths from COVID-19, which varies across borders resulting in inconsistencies among reports [5]. For example, countries such as China and Bangladesh, have changed the definition of confirmed cases or recoveries during the on-going pandemic [6, 7]. Moreover, leadership and political goodwill during such crisis play a crucial role in data collection, testing quality and country-wide coverage [8].

## Testing coverage

Testing coverage in the Indian subcontinent has yet to reach the peripheral areas. The government testing facilities are mostly free but are consequently overwhelmed with backlogs, whereas the costs of private tests are well out of the reach of most people [9]. Particularly with countrywide lockdowns and reduced patient transport facilities, it is hard to acquire free-public amenities as an observed 60% increase in extreme poverty in Bangladesh and 90% of people (around 400 million) working in the informal economy in India are at the risk of deeper poverty due to the lack of work during lockdowns [10, 11]. In such scenarios, people living in urban slums or rural areas are likely to prioritise wage-earning activities meeting the heightened unmet basic needs in the midst of low economic flow considering the risks of COVID-19 infection instead of a 14-day post-test quarantine.

Furthermore, given that the testing centres are mostly located in metropolitan areas, the coverage is often centralised to a few locations. For example, as of 18 June 2020, 64.1% of all tests in Bangladesh were conducted in Dhaka district compared to 0.01% in Jessore district [12], and 38.3% tests in Pakistan were conducted in Sindh compared to 0.50% in Gilgit-Baltistan [13]. This decidedly undermines the coverage across these countries and limits the cases to few privileged cohorts challenging identification of the disease at the community level and under-representing cluster transmission for an extended period of time. The lack of testing facilities in peripheral areas requires samples to be transported over long distances in a limited timeframe, which could jeopardise the reliability of samples and test results. The presence of centralised laboratories in few locations contributes to inadequate risk assessments across the country and impacts subsequent decision making.

## Funeral statistics

The BBC has put forward an interesting idea of calculating the underreported deaths of COVID-19 using fatality data from previous years [14]. While this could be a way forward, the burden of diseases varies yearly. One particular example could be the expected decrease in road fatalities in 2020 and an increase in suicide rate due to the adverse effects on mental health from stress during lockdown. Similarly, as COVID-19 is not identical with regard to seasonal outbreaks such as measles or dengue in Bangladesh or wild polio in Pakistan, we cannot predict COVID-19 fatality rate from the mortality of the previous seasonal outbreaks, which is likely to lead to a dubious understanding of COVID-19 numbers in these countries. Instead the total increase in death count after adjusting for typical seasonal diseases observed in the previous years could provide a better crude estimate of the impact of COVID-19.

Another avenue of estimating some of the deaths by COVID-like symptoms is data from graveyards and crematoriums. In West Bengal, India, the number of bodies cremated is nearly seven times more than the typical rate, whereas in Dhaka and Narayanganj districts of Bangladesh have seen twice or three times more burials in May 2020 compared to March or April [15, 16]. Disease misclassifications based on the differentiation in information regarding COVID-19 mortality apart from its comorbidities across multiple sources can adversely impact comparative analyses. This can also undermine subsequent resource mobilisation and evidence-based decision making in generating appropriate COVID-19 management and response worldwide. Moreover, the deceased with COVID-like symptoms are often untested in these countries, which although is understandable considering the resource limitations, but again considerably undermines the overall death tally.

## Spread among prominent public figures

Another method of scrutinising the underreporting of cases is to assess the data of frontline workers since they are more likely to be tested alongside the politicians. As of June 2020, 3.28%, 2.88% and 1.45% of COVID-19 fatalities in Bangladesh, Afghanistan and Pakistan were health workers respectively, whereas they were 0.37%, 0.55% and 0.50% in the USA, UK and Italy, respectively. These indicate that the health workers lacked adequate protective gear and knowledge about infection prevention and control measures in the ISC during the preparation phase as well as the fact that they were likely to be over-represented in the tests conducted, leading to an overall underreporting.

As of 19 June 2020, a total of 14 members of the Bangladesh parliament out of 350 had tested positive with two fatalities and over 100 staff of the parliament secretariat, which resulted in a truncated budget discussion in the parliament [17]. Moreover, 8.1% of the COVID-19 infected belongs to the Bangladesh police with 28 fatalities so far [18]. Their testing is expectedly prioritised and the rapid increase in these numbers indicates that community transmission has been severely underreported in the ISC.

## Cultural differences

In the ISC, people over 60 generally do not go out of home much, and often their external visits are limited to their familial circle [19]. Furthermore, a considerable number of women are homemakers [20]. Thus, a large portion of the society is used to staying at home, where able males mostly go out for work. While these scenarios are gradually changing, it could partially explain the slower transmission in ISC compared to the developed nations where all household members are more likely to go out increasing the speed of infection.

Social dogma regarding the COVID-19 is also playing a role in these nations. People in Pakistan and India are typically religious. There exist concerns among them that some of the funeral rituals, such as bathing the deceased, cannot be performed if they died of COVID-19 which has created public resentment towards testing [15]. Furthermore, neighbourhood protests were observed in Bangladesh where locals denied the COVID-19 deceased to be buried in their local graveyards [21]. Thus, comparison of case prevalence and fatalities across countries need to consider the cross-cultural and demographic factors.

## Biased findings?

There exists a major cause for concern regarding the data quality in the ISC. The subsequent use of these data in their raw form could lead to biased findings [22]. Davies *et al*. rightly found that younger age could be a protective factor in LMICs [23]; however, it is still too early to extrapolate any generalised conclusions. Non-random sampling has been conducted in the ISC, and their limited capacity forces them to test mostly the symptomatic individuals and foreign returnees from the high risked countries in the early days. Thus, statistical or epidemiological modelling might be statistically unprincipled with marginalised results when not taking into account the weaknesses of the data generating mechanism [24].

Some findings are developing on a regular basis. For example, in the early days of the pandemic, a hypothesis was shown to be ‘statistically significant’ that temperature is associated with the infection rate [25] without adequate information on true *R*_0_ value and its impact over COVID-19 transmission, which gave a misleading hope to politicians who used it to assure the general mass in Bangladesh leading to a sense of nationwide complacency [26]. Another assumption was that they are genetically immune to the coronavirus, or BCG vaccine might work as a protective factor [27]; however, the large death tolls of Bangladeshi expatriates in Saudi Arabia, Singapore and New York have evidently debunked it [28]. This false optimism has led to relaxation of social distancing policies in public transport and consideration of opening schools across the country [29, 30].

## Recommendations

**Fig. 1.**
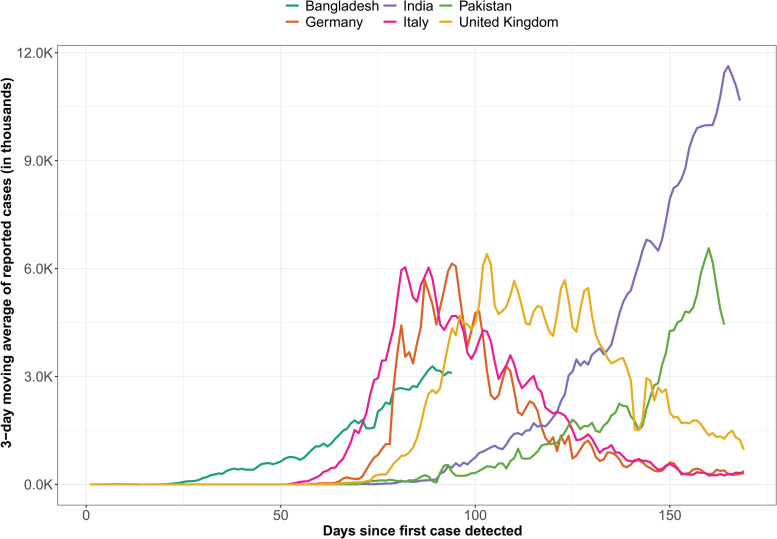
Distribution of reported COVID-19 cases in three higher income countries (UK, Italy and Germany) and three countries of ISC (Bangladesh, India and Pakistan) for the first 169 days (until 16 June 2020).

**Fig. 2.**
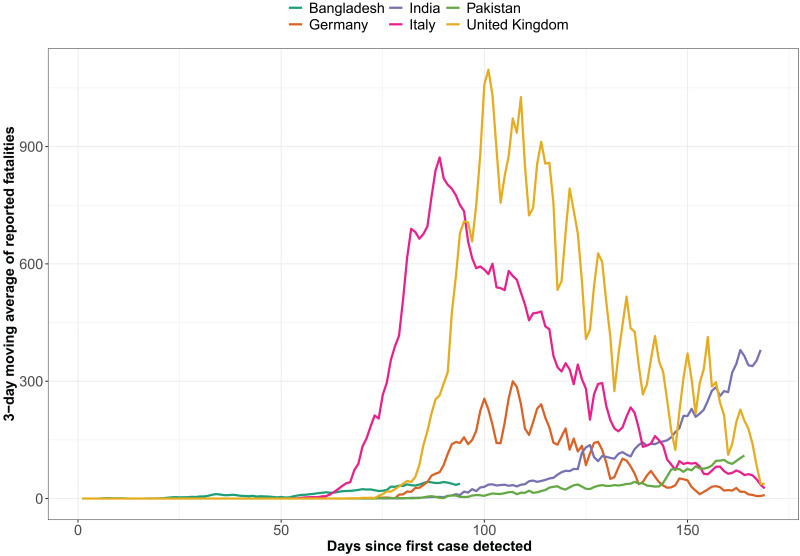
Distribution of reported COVID-19 fatalities in three higher income countries (UK, Italy and Germany) and three countries of ISC (Bangladesh, India and Pakistan) for the first 169 days (until 16 June 2020).

While it is inevitable that modelling with data from LMICs on COVID-19 would continue, a few cautions should be exercised:

- An appropriate definition of ‘death from COVID-19’ is essential before collapsing deaths from the COVID-like symptoms with the COVID-19 fatalities. For example, Bangladesh changed the definition of COVID-19 recovery a month after the detection of the first case [7].
- For validating the COVID-19 fatality scores of a region, specific mortality causes of comorbid conditions such as respiratory and cardiovascular complication or communicable diseases representing similar manifestation of symptoms linked to COVID-19 could be coded to calibrate from the total deaths during the pandemic period. However, this also needs to consider the seasonal outbreaks of diseases in specific regions.
- Comparison among seemingly random countries based on convenience or data availability might lead to a systematic bias. A comparative assessment on countries with similar testing coverages, analogous socioeconomic context, close geographical borders (e.g. EU or ISC), cultural resemblances and context-specific priorities with homogeneous health systems might be more insightful.
- Contrasting country-wise performances and COVID-19 infection timeline, where the COVID-19 prevalence curve has started to flatten with countries that are yet to reach its peak (Figs 1 and 2), are unreasonable as true propensity of the pandemic is yet to be observed in LMICs such as ISC.
- Instead of modelling COVID-19 incidence rates across borders, cultures and demographics, this could be limited to regions with homogeneous attributes. Comparison between neighbourhoods in the same locality might be better suited for hypothesis testing on mortality and disease prevalence, with the utmost care in avoiding narratives that might mislead the public opinion.
- Validating the COVID-19 official data with random sampling, hospital data, disease burden trends and local news outlets could account for some of the underreporting biases.

With thousands of PhD dissertations and research articles developing the evidence-base are expected on COVID-19 in years to come, it is imperative that the data validity is constantly questioned, and cross-border comparisons are routinely scrutinised given the definition of fatalities from COVID-like symptoms and quality of non-random data vary worldwide.

## Data Availability

The data that support the findings of this study are openly available at https://www.ecdc.europa.eu/en.
